# A stepped-wedge randomised-controlled trial assessing the implementation, impact and costs of a prospective feedback loop to promote appropriate care and treatment for older patients in acute hospitals at the end of life: study protocol

**DOI:** 10.1186/s12877-020-01660-2

**Published:** 2020-07-29

**Authors:** Xing J. Lee, Alison Farrington, Hannah Carter, Carla Shield, Nicholas Graves, Steven M. McPhail, Gillian Harvey, Ben P. White, Lindy Willmott, Magnolia Cardona, Ken Hillman, Leonie Callaway, Adrian G. Barnett

**Affiliations:** 1 Faculty of Health, School of Public Health and Social Work, Queensland University of Technology (QUT), Kelvin Grove, Queensland Australia; 2 Australian Centre for Health Services Innovation, Queensland University of Technology (QUT), Kelvin Grove, Queensland Australia; 3 Duke-NUS Postgraduate Medical School, National University of Singapore, Singapore, Singapore; 4 Adelaide Nursing School, University of Adelaide, Adelaide, South Australia Australia; 5 Australia Centre for Heath Law Research, Faculty of Law, Queensland University of Technology, Brisbane, Queensland Australia; 6 Gold Coast University Hospital, Southport, Queensland Australia; 7 Institute for Evidence-Based Health Care, Bond University, Robina, Queensland Australia; 8 Simpson Centre for Health Services Research, South West Sydney Clinical School, University of New South Wales, Liverpool, New South Wales Australia; 9 Faculty of Health, Queensland University of Technology, Kelvin Grove, Queensland Australia; 10 Faculty of Medicine, University of Queensland, Herston, Queensland Australia; 11 Royal Brisbane and Women’s Hospital, Herston, Queensland Australia

**Keywords:** End-of-life care, Geriatrics, High-risk, older population, Risk assessment, Intensive care, Medical futility, Non-beneficial treatment, Prospective feedback loop intervention, Stepped-wedge cluster randomised trial

## Abstract

**Background:**

Hospitalisation rates for the older population have been increasing with end-of-life care becoming a more medicalised and costly experience. There is evidence that some of these patients received non-beneficial treatment during their final hospitalisation with a third of the non-beneficial treatment duration spent in intensive care units. This study aims to increase appropriate care and treatment decisions and pathways for older patients at the end of life in Australia. This study will implement and evaluate a prospective feedback loop and tailored clinical response intervention at three hospitals in Queensland, Australia.

**Methods:**

A stepped-wedge cluster randomised trial will be conducted with up to 21 clinical teams in three acute hospitals over 70 weeks. The study involves clinical teams providing care to patients aged 75 years or older, who are prospectively identified to be at risk of non-beneficial treatment using two validated tools for detecting death and deterioration risks. The intervention’s feedback loop will provide the teams with a summary of these patients’ risk profiles as a stimulus for a tailored clinical response in the intervention phase. The Consolidated Framework for Implementation Research will be used to inform the intervention’s implementation and process evaluation. The study will determine the impact of the intervention on patient outcomes related to appropriate care and treatment at the end of life in hospitals, as well as the associated healthcare resource use and costs. The primary outcome is the proportion of patients who are admitted to intensive care units. A process evaluation will be carried out to assess the implementation, mechanisms of impact, and contextual barriers and enablers of the intervention.

**Discussion:**

This intervention is expected to have a positive impact on the care of older patients near the end of life, specifically to improve clinical decision-making about treatment pathways and what constitutes appropriate care for these patients. These will reduce the incidence of non-beneficial treatment, and improve the efficiency of hospital resources and quality of care. The process evaluation results will be useful to inform subsequent intervention implementation at other hospitals.

**Trial registration:**

Australia New Zealand Clinical Trial Registry (ANZCTR), ACTRN12619000675123p (approved 6 May 2019),

## Background

The older population is increasing worldwide as a result of longer life expectancy and lowered fertility rates [1]. Older persons typically have increased frailty, chronic health conditions and multiple morbidities [2, 3]. While health care use and expenditure in this population is variable, health care expenditure is markedly higher at the end of life [2, 4].

There are challenges to caring for this older population in acute care settings. Specifically, clinicians, patients and families grapple with tensions between the limits of medicine, subjective judgements about beneficence, and economic and clinical imperatives to provide appropriate and quality patient care [5]. Clinicians providing end-of-life care, who are often tasked with preparing patients and families for a transition to palliative and supportive care pathway [6], frequently experience barriers to these discussions [7, 8]. This leads to an increase in non-beneficial treatment, causes moral distress to clinicians, and prolongs or increases patient suffering [9]. A systematic review of 38 international studies found 33 to 38% of patients received non-beneficial treatment at the end of life [10].

These barriers have been broadly categorised as arising from clinician factors, hospital factors and patient factors [7, 11–14]. Addressing these factors is challenging, especially in large, complex acute care settings. Evidence exists for interventions to reduce non-beneficial treatment outside of acute hospitals [15–18], and an intervention study has been done in the Intensive Care Unit (ICU) setting in the United States [19]. There is no published research in Australia evaluating an intervention to reduce non-beneficial treatment at the end of life in hospitals.

As with other developed countries, Australia’s health care system faces urgent challenges from an ageing population. This population is more likely to be hospitalised than in the past, with hospitalisation rates for people aged over 85 years increasing by 35% for women and 48% for men in the decade to 2011 [20]. The end-of-life phase in Australia is becoming an increasingly medicalised experience with 54% of deaths occurring in hospital [21]. A retrospective study [22] of three Australian hospitals in 2012 identified 12.1% (range 6.0 to 19.3%) of end-of-life admissions received non-beneficial treatment. These admissions received non-beneficial treatment for an average of 15 days including 5 days spent in the ICU. This same study estimated an annual national health system cost of $A153 million due to non-beneficial bed days.

The Intervention for Appropriate Care and Treatment (InterACT) study will implement a prospective, tailored feedback loop intervention in three acute hospitals in Queensland, Australia. We will evaluate whether the intervention improves care outcomes for older patients, specifically to increase appropriate care and treatment pathways, and reduce the incidence of non-beneficial treatments. This study will use two validated tools concurrently to prospectively identify patients at the end of life where curative and life-sustaining interventions may be non-beneficial. The Criteria for Screening and Triaging to Appropriate aLternative Care (CriSTAL) tool was developed to identify elderly patients in the last months of life [23–25]. The Supportive and Palliative Care Indicators Tool (SPICT™) can be used to identify patients at risk of deteriorating and dying [26] with recent studies reporting an association between a SPICT-positive result and one-year mortality [27, 28].

Patient screening with both tools will be the first step in the prospective feedback loop intervention. Providing feedback to clinical teams aims to increase clinician awareness of their patients’ risk profile, directly addressing some of the known clinician and hospital factors for non-beneficial treatment [29–31]. A tailored clinical response to this information will be determined and implemented by the clinical teams, with support from a hospital executive advisory group.

The primary objective of the study is to determine the impact of a tailored clinical team feedback loop intervention on outcomes related to appropriate care and treatment at the end of life. The extent of change in health service use and costs will also be estimated. A second objective is to conduct a process evaluation to assess implementation, mechanisms of impact, and contextual barriers and enablers of the feedback loop intervention.

## Methods/design

### Study design

**Fig. 1:**
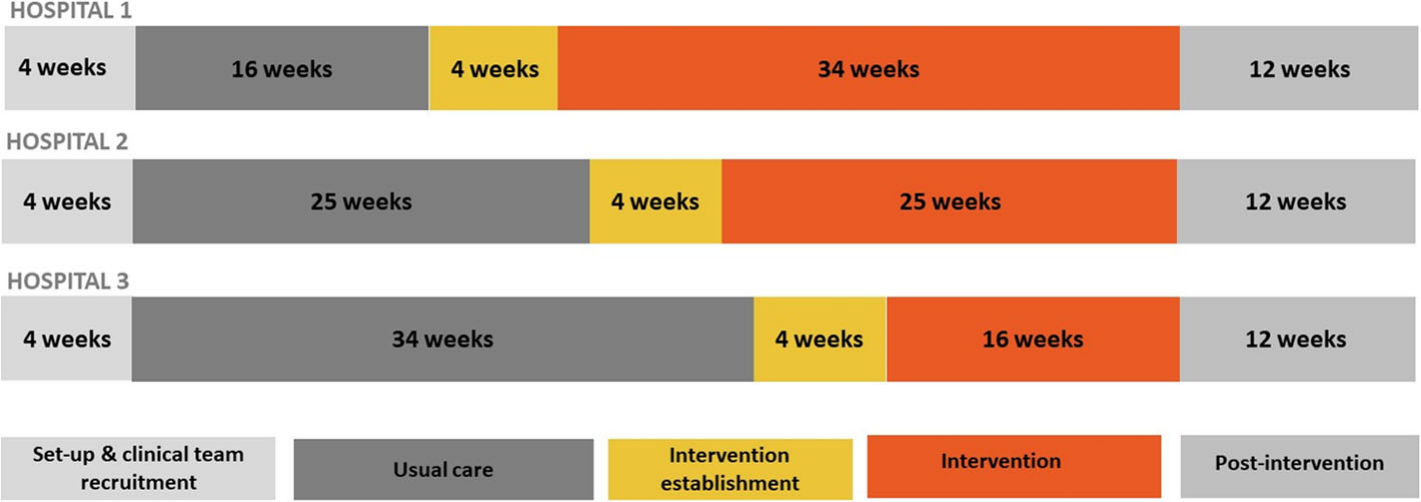
Stepped-wedge study design in three hospitals with seven teams per hospital for the InterACT trial

This is a randomised controlled trial using a multi-centre stepped-wedge randomised design (Fig. 1). Five stages will be sequentially rolled-out across the three hospitals over 70 weeks: site preparation; usual care exposure; intervention establishment; intervention exposure; and post-intervention. The timing of the one-way crossover from usual care to intervention establishment will be randomly allocated.

### Study setting and population

This trial will be undertaken at three large, acute Queensland teaching hospitals. We will aim to enrol seven clinical teams at each hospital. In consultation with the local hospitals’ executive advisory group, we will purposively sample from acute-care clinical teams and medically-oriented clinical specialities that have a regular number of patient admissions ≥75 years. This supports trialling the intervention in clinical teams that routinely have an older patient population who are more likely to receive non-beneficial treatment.

For inclusion, clinical teams must:

- be an established clinical team unit or specialty that routinely admits patients within the hospital
- include at least one nominated lead specialist consultant
- include at least one registrar and affiliated clinical nurse consultant or nurse unit manager
- have a clinical team structure and admission pattern typical of the hospital
- have a consistent history of admitting patients aged 75 years or over during the previous year
- participate in an information session with the project team.

Excluded clinical teams will additionally include those already implementing interventions or initiatives related to reducing non-beneficial treatments for older patients, and those from the emergency department, any Intensive Care Units (ICUs), mental health units, and non-acute care. The excluded specialities likely have different clinical and treatment focus which would require a different implementation plan.

At each hospital, enrolled clinical teams, executive advisory group members and site study team members will be recruited for the process evaluation. Site study team members will include hospital employees who have a role in supporting the implementation of the trial or in data collection, as determined in the site preparation phase, and may include the nurse auditor, site coordinator, and palliative care team.

### Intervention

**Fig. 2:**
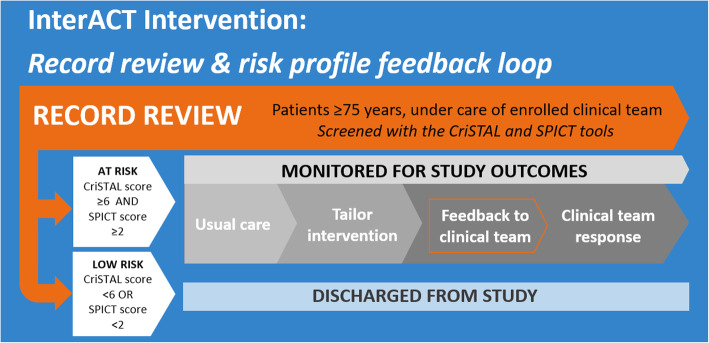
Schematic of the InterACT intervention to be implemented

The study intervention (InterACT) is a prospective feedback loop to clinical teams with tailored clinical response, based on the outcomes of a patient record review (or screening) process using the CriSTAL and SPICT tools. The intervention process is shown in Fig. 2.

For the purpose of this study, patients are considered to be in the at-risk population if they were identified to be *high-risk CriSTAL* and *SPICT-positive*. Patients are classified as *high-risk CriSTAL* (at high risk of death within 3 months) if they have a CriSTAL score of 6 or greater. Patients with a SPICT general indicator score of 2 or greater are considered *SPICT-positive* (presence of indicators of potential deterioration within 12 months).

Site based trained auditors will review patient records of new admissions to participating clinical team twice weekly to identify patients in the at-risk population. Record review data will be collected using an electronic form in REDCap [32].

### The feedback loop

In the intervention exposure phase, the study team will provide feedback based on the record review results to individual clinical team nominees after each screening episode. The study team will develop computer scripts to automate the processes of exporting the data from REDCap into R [33], processing the data and generating the feedback report. The project manager will provide the feedback report to clinical team nominees in an agreed format. The feedback report process includes email, text or database log-in (or a combination of).

This feedback is intended to flag that a patient is in the at-risk population, and to act as a stimulus to review patient care activities and pathways [30, 34]. Clinician expertise and autonomy will not be questioned in the feedback, nor will judgements be made about the likelihood of non-beneficial treatment. Examples of potential clinician response to the feedback include palliative care referral, multidisciplinary team review, advance care planning consultation, and patient/family meetings.

### Implementation framework

We will use the Consolidated Framework for Implementation Research (CFIR) [35] to inform the implementation and process evaluation of the InterACT study intervention. The intervention will be fully described in a CFIR-based implementation toolkit that identifies what parts of the feedback loop intervention will be fixed, what will be flexible, and the associated degree of flexibility.

The toolkit will support the local tailoring of the intervention to reflect hospital context, clinical team structure and workflows. The project team, study team, hospital executive advisory group and enrolled clinical teams will collaboratively develop and pilot an effective feedback loop and associated clinical team response during the intervention establishment phase.

### Monitoring and evaluating implementation

Process evaluation is an essential part of designing and testing complex interventions [36]. The real-world setting and length of this trial will require a pragmatic approach to fostering intervention adherence, reach and fidelity. The project team will systematically monitor the implementation process as part of the process evaluation using templates and approaches based on the CFIR constructs. This embedded approach will provide direct support for implementing the InterACT study intervention and will inform understanding of how the implementation process contributes to the study outcomes.

### Study outcomes

The study outcomes and outcome measures are described in Table 1. Outcomes 1 to 9 are for patients admitted under the enrolled clinical teams and identified to be in the at-risk population following screening with the CriSTAL tool and SPICT. The measurement tools used are summarised online [37].

**Table 1:** Project outcomes and outcome measures for the InterACT study

Outcome label	Outcome	Outcome measure
**Impact outcomes**		
Primary outcome (Outcome 1)	Proportion of patients with one or more Intensive Care Unit (ICU) admissions	ICU admissions during the current hospital stay from the date first recorded as *high-risk CriSTAL and SPICT-positive*.
Outcome 2	Length of hospital stay and discharge outcome	Length of hospital stay, with the transition endpoints of ‘discharged alive’ and ‘death in hospital’, from the date first recorded as *high-risk CriSTAL and SPICT-positive.*
Outcome 3	Time to hospital re-admission	The time in days to re-admission to any Queensland public hospital for re-admissions within 12 weeks from the date of discharge.
Outcome 4	Time to first documented indications of clinician-led care review discussion	The time in days from the date first recorded as *high-risk CriSTAL and SPICT-positive* to documentation of a clinician-led care review activity. The type of care review activity (reduce/cease active treatment, increase comfort care, continue active treatment) and indications of family conflict will also be recorded.
Outcome 5	Time to first care directive measure	The time in days from the date first recorded as *high-risk CriSTAL and SPICT-positive* to documentation of any care directive (including discussion outcomes, advance care plan, statement of choices, acute resuscitation plan). The type of care directive will also be recorded.
Outcome 6	Time to first palliative care referral	The time in days to first documented palliative care referral from the date first recorded as *high-risk CriSTAL and SPICT-positive* during the current hospital stay.
Outcome 7	Time to first medical emergency call	The time in days to first medical emergency call during the current hospital stay.
**Health care resource use and cost outcomes**		
Outcome 8	Changes in admission/hospitalisation and treatment costs	Costs of treatment will be taken from routinely collected information and will begin accumulating from the date first recorded as *high-risk CriSTAL and SPICT positive*. This ensures that treatment costs reflect only those costs that relate to care provided at the end-of-life phase. All costs will be stratified by the acute and palliative care phases so that any changes resulting from different treatment pathways can be identified.
Outcome 9	Cost of implementing the prospective feedback loop intervention	The cost of implementing the study intervention will be measured by the duration and unit costs of staff time associated with completing direct trial activities (including document review and clinical team feedback activities).
**Process outcomes**		
Extent and fidelity of intervention implementation, impact, and contextual barriers and enablers of the feedback loop intervention		

We chose ICU admissions as the primary outcome as the at-risk population is less likely to benefit from ICU admission, and it is a commonly used proxy for non-beneficial treatment in hospital [10]. That said, this is a complex trial and the overall impact of the intervention will be more appropriately represented through consideration of all outcomes listed.

### Study procedures

#### Recruitment, enrolment and withdrawal

The project manager will invite eligible clinical teams to participate at routine meetings in the hospitals during the recruitment period. The project manager, an executive sponsor and site-based study coordinator will explain the study and what is involved. All interested clinicians will have access to the participant information sheets and full study protocol [37].

Clinical teams will be enrolled by the project manager. A nominated lead clinician will provide agreement on behalf of their clinical team. As the unit of enrolment is the clinical team, if an individual clinician participant, including the lead clinician, withdraws from the study, data collection and clinical team participation will continue unchanged.

Once enrolled, clinical team participation will only be discontinued if ongoing participation is untenable or negatively impacting patient care. The trial will only be discontinued if a regulatory body, funding body, or ethics committee judges it necessary for medical, safety, regulatory, or other reasons consistent with applicable laws, regulations and good clinical practice.

#### Randomisation, allocation and blinding

All three hospitals will receive the intervention, with the timing randomised. A statistician investigator (XJL) will be responsible for computer generation of the trial timing randomisation as per Fig. 1 and the intra-hospital clinical team identifiers. These identifiers will help anonymise the data storage and reporting.

Given the nature of the intervention and trial design, it is not possible to blind the clinical teams to the intervention. The intervention exposure phase commencement date will be concealed from the hospital teams during the site preparation and clinical team recruitment phase. Hospital teams will be notified of their intervention establishment and intervention exposure start dates about 8 weeks prior to allow time to plan for the intervention establishment phase activities.

#### Data collection

Site-based trained nurse auditors will prospectively collect identifiable patient data during record screening using an online form in REDCap. The auditors will review patient records of all new admissions to participating clinical teams twice weekly to identify patients in the at-risk population. At-risk patients will be passively monitored at subsequent screening times if they have not been discharged or transferred to a different facility. Auditors will record if and when these patients had documented indications of clinician-led care review discussion, care directive measures, and palliative care referral. Data custodians will link outcome data for patients at the end of the intervention exposure period.

Members of the clinical team and the site-based study team will be asked to complete monitoring documents, including capturing time spent on intervention activities, to assess adherence to and fidelity of the intervention.

Process evaluation data will be collected using templates based on the CFIR. Clinical team members will be invited to participate in group or individual interviews after the pilot in the intervention establishment phase, during the intervention exposure phase, and during the post-intervention phase. The hospital advisory group and site-based study teams will also be asked to voluntarily participate in a group or individual interview about the implementation process during the post-intervention phase.

Additional details on the data items, collection methods and schedule, and data management are provided online [37].

### Data analyses

#### Sample size and statistical power

Simulation-based sample size calculations [38] were used to determine an adequate sample size to detect the change associated with the intervention using the primary outcome. Information used in the simulation, including the effect size, were derived from a previous study estimating the incidence and impact of non-beneficial treatment in three Queensland tertiary public hospitals [22].

Weekly identification of at least three at-risk patients in each of the 21 clinical teams across the 3 hospitals will give a statistical power of 95% to detect a reduction in the proportions of patients with ICU admissions from 0.20 in the usual care exposure period to 0.113 in the intervention exposure period. The sample size calculations were based on a 5% statistical significance level for a two-sided test, within-ward correlation of 0.1, and a stepped-wedge design as shown in Fig. 1. We performed a sensitivity analysis with an alternative within-ward correlation of 0.01 and estimated the power to be 79.3%.

#### Statistical analyses

We will use R for data management, modelling and graphics [33]. We will make all our R code publicly available via GitHub. We will use EQUATOR guidelines to write-up our results, using the CONSORT guidelines for randomised trials, the extension for stepped-wedge trials [39, 40]. Additional details of the statistical analysis plan are provided online [37].

#### Analysis of primary outcome

The primary outcome, proportion of patients with at least one ICU admission, will be analysed using a mixed effects Binomial regression. The key variable is the timing of the switch from usual care exposure to intervention exposure phase. The intervention timing regression coefficient will be treated as a random slopes term to account for potential differences in intervention effects across clinical teams. A linear time covariate will be included to capture a potential calendar time trend. The model will also adjust for likely predictors of patient age and sex. The model will include a random intercept for each enrolled clinical team to account for any underlying differences in the proportion of patients with ICU admissions between the teams.

#### Analyses of secondary outcomes

Competing-risk proportional hazards survival models will be used to analyse time-to-event outcomes during patient hospitalisations (outcomes 2, 4, 5, 6 and 7). Hospital discharge and in-hospital deaths are treated as competing risks. Use of the competing risk survival model provides an estimate of the intervention effect which appropriately accounts for the competing and time-varying nature of discharge and death on the outcomes [41]. The models will have a binary variable indicating if patients were in the intervention exposure phase. The survival analysis will adjust for likely predictors of patient age, sex and time spent in hospital for identified hospital episode prior to admission to enrolled clinical team. The survival analysis will stratify by clinical teams to control for consistent differences between teams. Cumulative incidence curves will be used to compare the event rates over time between the usual care exposure and intervention exposure phases.

We will use a proportional hazards survival model to investigate the difference between usual care and intervention exposure phases in the time to re-admission within the first 12 weeks after index discharge (outcome 3). Patients who died after leaving hospital or were not re-admitted within the 12 weeks will be censored. Clinical teams will be included as strata.

Data on the health services used by patients during their hospital admission will be retrieved from the hospitals’ clinical costing database to evaluate changes in admission and treatment costs (outcome 8). Any costs that occurred before the patient was identified to be in the at-risk population will be excluded. Statistical distributions will be fitted to describe variability in all cost parameters. The key measure of cost will be the average cost per patient. This will be presented alongside 95% bootstrap confidence intervals to estimate the uncertainty in this average.

The costs of implementing the intervention (outcome 9) will be measured by the staff time associated with collecting, interpreting and providing the feedback, and for all activities required to establish the intervention. The economic opportunity costs of healthcare workers’ time will be valued by Queensland Health wage rates. Quantities and types of consumables and incidentals used will be valued in dollar terms by market price. The outcome will be reported as the estimated total cost of the intervention and bootstrapped 95% confidence interval.

#### Process evaluation data analysis

Interview notes and transcripts, and monitoring and field records will be entered into NVivo [42] and analysed qualitatively. The CFIR framework constructs – intervention characteristics, individual characteristics, inner setting, outer setting and process – will be used as an initial coding framework for the data [35]. Within these broad constructs, thematic analysis will be used to identify and compare patterns in the data within and across intervention sites [43]. Data that are distinct from the CFIR constructs will be analysed inductively, again using a thematic approach. Coding and cross-checking of data will be undertaken independently by two research team members. Differences will be discussed and, where necessary, additional members of the research team will be engaged to reach agreement. Analyses will be synthesised to produce a narrative account of the processes of implementation in relation to observed outcomes.

## Discussion

The emphasis of this study is to support clinicians to recognise the potential for non-beneficial treatment at the end of life using a feedback loop that relays objective information about risk profiles in relation to death and deterioration. The tailored clinical response promotes communication, engagement and awareness. Audit and peer feedback approaches have been considered promising for over two decades in areas such as improving the accuracy of clinical documentation [44] and potentially assisting in clinician’s behaviour change [45].

The expected benefits are improved patient outcomes in terms of reduction in non-beneficial treatments following the clinical team response to the patient record review data, specifically the CriSTAL and SPICT scores and indicators. This should facilitate planning and delivering appropriate care and treatment to those patient groups. Cost savings may be observed due to the prevention of non-beneficial treatments. In addition, the qualitative process evaluation will identify key factors to the intervention’s implementation and performance, as well as the potential to be implemented at other hospitals, in line with best-practice recommendations for these interventional trials [30, 46].

The stepped-wedge design used has several strengths. The incremental roll-out of the stepped-wedge design is practical to implement, mimics how the intervention might be implemented in practice at other hospitals [47] and is well-suited to the evaluation of health service delivery interventions [43]. This design allows the study team to work with each hospital during the intervention establishment phase. Each hospital contributes data to both the control and intervention conditions, mitigating risk associated with comparing heterogeneous hospitals. This crossover also means that temporal effects can be studied [48] with more efficiency than other cluster designs [49]. The risk of contamination between sites and teams is minimised by geographical separation of sites and simultaneous crossover of clinical teams within the same site. The main drawbacks of this design are the potential risk of secular trends unrelated to the intervention exposure, and risk of unequal exposure to seasonal trends. These risks were taken into consideration in the statistical analysis plan.

There are economic and clinical imperatives to reduce non-beneficial treatments within the Australian healthcare system. By concurrently completing a process evaluation of the study intervention, we will identify the barriers and enablers to using objective data to promote appropriate patient care pathways. This research will be useful for implementation of this intervention in other hospital settings.

### Study status

The protocol version is 6.0 (28 January 2020), which has been adapted to suit the formatting requirements of the journal. The trial is prospectively registered with the Australia New Zealand Clinical Trial Registry (ANZCTR), ACTRN12619000675123p (Registration date: 6 May 2019).

All ethical and governance approvals are in place at each of the participating hospitals. The trial component started recruitment in February 2020, with an expected end date of August 2021.

## Data Availability

At the end of the study, final non-identifiable data sets will be deposited in QUT’s Research Data Storage System. In line with publication embargoes and requirements, we will generate a document object identifier for each non-identifiable data set, and make this record publicly accessible.

## Abbreviations

ANZCTR: Australian and New Zealand Clinical Trial Registry
CFIR: Consolidated Framework for Implementation Research
CONSORT: Consolidated Standards of Reporting Trials
CriSTAL: Criteria for Screening and Triaging to Appropriate aLternative Care
HREC: Human Research Ethics Committee
ICU: Intensive Care Unit
InterACT: Intervention for Appropriate Care and Treatment
GCHHS: Gold Coast Hospital and Health Service
GCUH: Gold Coast University Hospital
MET: Medical Emergency Team
MNHHS: Metro North Hospital and Health Service
QUT: Queensland University of Technology
RBWH: Royal Brisbane and Women’s Hospital
SPICT: Supportive and Palliative Care Indicators Tool
SPIRIT: Standard Protocol Items: Recommended for Interventional Trials
SSA: Site Specific Assessment
TPCH: The Prince Charles Hospital
